# Dexmedetomidine and Apigenin Combination Mitigates Memory Deficits Caused by Microglial Activation and Hippocampal Neurogenesis Impairment in Methotrexate‐Induced Chemo‐Brain in Rats

**DOI:** 10.1155/np/9434180

**Published:** 2026-05-11

**Authors:** Einas M. Yousef, Omar Mohsen Eldemerdash, Mahmoud A. Senousy, Mohamed Taha, Haidy Mohammed, Ismail Mohamed Elshaffei

**Affiliations:** ^1^ Department of Anatomy and Genetics, College of Medicine, Alfaisal University, Riyadh, 11533, Saudi Arabia, alfaisal.edu; ^2^ Department of Histology and Cell Biology, Faculty of Medicine, Menoufia University, 3251, Shebin Elkom, Menoufia, Egypt, menofia.edu.eg; ^3^ Department of Biochemistry, Faculty of Pharmacy, Misr International University (MIU), Cairo, Egypt, miuegypt.edu.eg; ^4^ Department of Molecular Biochemistry, Institute of Biochemistry, Universität Stuttgart, Stuttgart, D-70569, Germany, uni-stuttgart.de; ^5^ Department of Biochemistry, Faculty of Pharmacy, Cairo University, Cairo, 11562, Egypt, cu.edu.eg; ^6^ Department of Biochemistry, Faculty of Pharmacy and Drug Technology, Egyptian Chinese University, Cairo, 11786, Egypt; ^7^ Department of Internal Medicine, Faculty of Medicine, Cairo University, Cairo, 12613, Egypt, cu.edu.eg

## Abstract

Dexmedetomidine (Dex), an α2‐adrenergic receptor agonist, and apigenin (Api), a naturally occurring bioflavonoid, have recently shown neuroprotective effects in experimental models of methotrexate (MTX)‐induced neurotoxicity; however, the impact of their combined administration has not yet been elucidated. This study investigated the effects of Dex on microglial activation, neuroinflammation, and apoptosis, and the role of Api in hippocampal neurogenesis, along with its combined effects via the miR‐15a/ROCK‐1/ERK1/2/CREB/BDNF signaling cascade. Male Sprague Dawley rats were randomly assigned into: a normal control group, Dex and Api‐control groups, which received Dex or Api daily for 30 days, an MTX‐only group that received MTX and leucovorin (LCV), Dex or Api cotreated groups received either Dex or Api with MTX and LCV, and a Dex + Api cotreated group received both drugs with MTX and LCV. Dex improved cognitive function by attenuating microglial activation. Api mitigated hippocampal neurogenesis, as shown by decreased Ki‐67 and doublecortin (DCX) expression. Compared with Dex or Api alone, the combined treatment had more favorable effects on novel object recognition (NOR), miR‐15a, ROCK‐1, ERK1/2, histopathological changes, and Ki‐67 neurogenesis marker over Api or Dex monotherapy, and improved CREB/BDNF signaling over Dex alone. However, no additional favorable effects on microglial activation, redox balance, IL‐1β, or apoptosis were observed. The combination of Dex and Api exhibits more pronounced neuroprotective effects by potentiating modulation of the miR‐15a/ROCK‐1/ERK1/2/CREB/BDNF signaling cascade, improving cognitive behavior, and enhancing hippocampal neurogenesis.

## 1. Introduction

Chemotherapy is known to be associated with numerous systemic and neurological adverse effects, especially in pediatric patients, including chemotherapy‐induced cognitive impairment (CICI), commonly referred to as “chemo‐brain” [1]. A considerable proportion of pediatric cancer survivors develop cognitive deficits, memory issues, and neuromuscular dysfunction during chemotherapy [2, 3]. Although this impairment can be experienced mostly during therapy, the cognitive dysfunction detected in acute lymphoblastic leukemia (ALL) survivors can persist for years [4]. Methotrexate (MTX) can be used alone or in conjunction with other medications to treat rheumatoid arthritis and several neoplasms, including meningeal leukemia and ALL [5, 6]. While it is challenging for MTX to cross the blood–brain barrier (BBB), a high dosage of MTX can have neurotoxic effects [7]. MTX‐induced neurotoxicity elicits memory deficits most often in pediatric patients receiving ALL treatment, with reported prevalence rates ranging from 9% to 53% [8–11]. Recent studies suggest that MTX‐induced neurotoxicity involves microglial activation, neuroinflammation, and impaired hippocampal neurogenesis, leading to deterioration of spatial working memory and cognitive impairments [12, 13].

The hippocampus is critically involved in learning, memory, and cognitive processing, and it supports ongoing neurogenesis even throughout adulthood [14]. In adult mammals, hippocampal neurogenesis takes place in the subgranular zone of the dentate gyrus and involves tightly regulated proliferation, differentiation, and synaptic integration of neural progenitor cells, processes highly sensitive to inflammation, oxidative stress, and neurotrophic signaling [15]. Microglia play a critical role in maintaining the neurogenic niche by phagocytosing apoptotic newborn cells and regulating inflammatory signaling [16]. MTX has been shown to cause long‐term microglial activation and neuroinflammation, thereby disrupting the neurogenic niche and contributing to long‐term cognitive dysfunction [17]. Furthermore, MTX has been shown to disrupt the myelination process and to reduce the number of oligodendrocyte precursor cells [17]. The molecular pathway of MTX‐induced microglial activation was unclear until recently studied [12, 13].

The cAMP‐response element‐binding protein (CREB) is a transcription factor whose kinase‐inducible domain is activated by the protein kinase A (PKA) and the extracellular signal‐regulated kinase (ERK1/2) pathways [18]. Phosphorylated CREB (p‐CREB) promotes transcription of cAMP‐responsive genes, which encode vital proteins that regulate neurogenesis, the DNA damage response, cell cycle, and cell death. Notably, brain‐derived neurotrophic factor (BDNF) is a cAMP‐responsive gene that is impacted by p‐CREB [19, 20]. As one of the most significant neurotrophins, BDNF is strongly linked to the differentiation of hippocampal stem cells and the stimulation of neuron survival [21, 22]. Tyrosine kinase B receptor (TrkB) binding by BDNF activates neuroprotective signaling pathways, such as the ERK1/2/CREB pathway, which contributes to BDNF upregulation [23–25]. A decline in BDNF and disturbed redox homeostasis are implicated in numerous neurodegenerative disorders, including cognitive impairment and Parkinsonism [26–29].

Rho‐associated protein kinase‐1 (ROCK‐1) negatively regulates ERK1/2 signaling and has been implicated in neuroinflammatory processes. Downregulation of ROCK‐1 activates the ERK1/2/CREB pathway, which in turn promotes increased cell viability and restricts the apoptotic process [30]. Numerous investigations have shown that increased ROCK‐1 expression is entangled in several neurodegenerative disorders, including Alzheimer’s disease (AD), by regulating autophagosome formation, neuroinflammation, and cytokine production and release by the microglia [31–33]. Upstream of ROCK‐1 is miR‐15a, a microRNA that regulates gene expression and has recently been associated with controlling neurogenesis and memory. Reduced expression of miR‐15a has been reported in both human and experimental mouse models of temporal lobe epilepsy and AD [34–36]. Indeed, miR‐15a negatively regulates ROCK‐1 expression in hippocampal neurons treated with the amyloid β (Aβ) fragment (Aβ25−35), resulting in enhanced cell viability and reduced apoptosis [30]. Furthermore, MTX‐induced downregulation of miR‐15a has been associated with increased ROCK‐1 activation and subsequent suppression of the ERK1/2/CREB/BDNF pathway [12, 13].

Dexmedetomidine (Dex), a potent α2‐adrenergic receptor agonist, has sedative, analgesic, and anxiolytic effects [37]. The neuroprotective action of Dex is mediated by promoting neurogenesis through activation of the ERK1/2 pathway and subsequent phosphorylation. This phosphorylation cascade leads to CREB activation and its translocation to the nucleus, ultimately stimulating BDNF gene expression [13, 38]. In the hypoxic‐ischemic brain damage model, Dex was shown to promote oligodendrocyte proliferation and neuron myelination [39]. Recently, reports have indicated that Dex can mitigate MTX‐induced cognitive impairment and promote hippocampal neurogenesis in rats. This is achieved by regulating miR‐15a and its target genes in the ROCK‐1/ERK1/2/CREB/BDNF pathway [13]. Nevertheless, the impact of Dex on microglial activation remains uncertain.

Apigenin (Api), a natural bioflavonoid, has antioxidative and anti‐inflammatory properties and displayed potential for the treatment of cancer and other serious illnesses [40, 41]. Api has been shown to inhibit microglial activation in a MTX‐induced neurotoxicity rat model by modulating miR‐15a and targeting the ROCK‐1/ERK1/2/CREB pathway [12]. However, the impact of Api on hippocampal neurogenesis and its potential interaction with Dex have not been fully elucidated.

Given the complex interplay between microglial activation and neuronal signaling pathways in MTX‐induced neurotoxicity, further investigation is needed to clarify their potential mechanistic relationships. This work assessed the impact of Dex on microglial activation and Api on hippocampal neurogenesis in an MTX‐induced chemo‐brain model. In addition, we evaluated whether combined Dex and Api treatment provides greater neuroprotective effects than monotherapy with respect to hippocampal neurogenesis, microglial activation, redox status, neuroinflammation, and apoptosis. These effects were investigated in relation to the miR‐15a modulation and its associated ROCK‐1/ERK1/2/CREB/BDNF signaling pathway.

## 2. Materials and Methods

### 2.1. Animals

One hundred and five male Sprague Dawley rats (4–5 weeks old and 150–200 g) were acquired from the animal facility of the Faculty of Pharmacy, Cairo University (Cairo, Egypt). Animals were housed at the Misr International University animal home in a controlled environment. A constant temperature of 25 ± 2°C, a humidity of 60 ± 10%, and a 12 h light/dark cycle were all maintained. Rats had free access to a standard pellet diet and water throughout the study.

The experimental procedures and animal model complied with the rules established by the Research Ethics Committee for Experimental and Clinical Studies, Faculty of Pharmacy, Cairo University, Cairo, Egypt (permit number: BC2944). The accommodations for the animals were made in compliance with the US National Institutes of Health “Guide for Care and Use of Laboratory Animals” (No. 85–23, revised 2011). Every attempt was made to lessen the pain and the number of animals used.

### 2.2. Drugs and Chemicals

Intravenously administered 50 mg vials of MTX were purchased from Mylan S.A., France. GPI, Egypt, was the supplier of leucovorin (LCV) (Calcifolinon, 50 mg/vial). We also purchased dexmedetomidine hydrochloride (PrecedexTM, 200 μg/vial) from Hospira, United States. The doses were diluted with sterile water just prior to injection. After being purchased from Swanson, USA, 50 mg capsules of Api were dissolved in 10 mL of 0.5% carboxymethyl cellulose (CMC) solution just before oral administration. CMC sodium chloride was acquired from TopChem in Egypt.

### 2.3. Experimental Design

**Figure 1:**
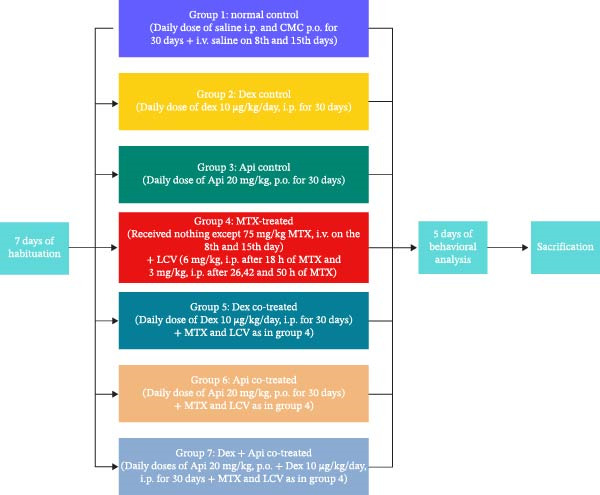
Experimental design.

Following a week of acclimatization, animals were allocated into seven groups (*n* = 15 each) at random. Group 1 (normal control) was administered daily dosages of CMC orally, normal saline was given intraperitoneally, and intravenous saline was given on 8th and 15th days of the experiment. The rats in group 2 (Dex control) were administered an intraperitoneal injection of Dex at a dose of 10 μg/kg daily for 30 days [42]. Group 3 (Api control) was administered a daily oral dose of Api at a concentration of 20 mg/kg dissolved in a 0.5% CMC solution for 30 days [41]. Group 4 (MTX‐treated) was given two doses of MTX (75 mg/kg) intravenously on days 8 and 15 [10, 29]. LCV was then given intraperitoneally in four doses: 6 mg/kg at 18 h followed by 3 mg/kg at 26, 42, and 50 h [11, 43]. LCV, a folate analog, was administered after each MTX injection to lessen the MTX toxicity, eliminate the lethal diarrhea and weight loss caused by MTX, and reduce the mortality rate. Rats in group 5 (Dex cotreated) were administered Dex at 10 μg/kg/day for a period of 30 days [42]. Additionally, the rats in this group were given MTX and LCV as described for group 4. Group 6 (Api cotreated) was administered MTX and LCV as in group 4 and daily oral dosages of Api (20 mg/kg) throughout the study for 30 days. Group 7 (Dex + Api cotreated) received both Dex and Api, along with MTX and LCV, as described for groups 5 and 6, respectively. The experimental design is represented in Figure 1.

All doses were selected based on previously published research. MTX (75 mg/kg) was injected through the lateral tail vein using a 27‐gauge needle under gentle restraint [10, 29]. The Api dose (20 mg/Kg, oral) was adopted from earlier work that proved its efficacy in mitigating cognitive impairment and upregulating CREB/BDNF in kindled mice [41]. While the Dex i.p. dose was selected based on the results of our previously published data following a pilot study [13]. Additionally, the i.p. doses of LCV were chosen based on prior research, including our pilot study [10, 13, 29]. All routes of administration were decided based on previous studies conducted on similar animal models and according to the available dosage forms in the market.

Ten rats from each group were chosen at random 30 days after the beginning of the model, and behavioral tests were conducted to evaluate the rats’ memory and cognitive abilities. The rats were anesthetized via inhalation of 3% isoflurane 24 h after behavioral testing, after which they were killed via cervical dislocation. The brain tissues were collected and promptly rinsed with ice‐cold saline. Samples were processed for hippocampal isolation from each brain side and stored at −80°C for subsequent biochemical analysis (*n* = 6) or promptly submerged in 10% formol saline for histological and immunohistochemical analysis (*n* = 4).

### 2.4. Behavioral Analysis

Novel object recognition (NOR) and Morris water maze (MWM) tests were chosen for behavioral analysis because they are available in the animal facility, are good at assessing memory and cognitive function, and can be automatically analyzed via Any‐Maze software (version 7.1, Stoelting Co, IL, USA).

#### 2.4.1. NOR Test

The NOR test was conducted at the end of the experimental period to assess recognition memory by evaluating the ability of rats to distinguish familiar from novel objects, a function closely linked to hippocampal integrity. The day prior to the examination (last day of the model), each rat underwent a process of habituation within an unoccupied open field arena (1 × 1 × 0.5 m) for a duration of 5 min. Each rat had two trials during the testing session: a choice trial and a familiarization trial. Behavioral performance was recorded and analyzed using ANY‐maze software. To eliminate any possible evidence, the arena and its contents were completely cleansed with a 70% ethanol solution following each trial.

During the time of familiarization in the experiment, every rat was subjected to a pair of identical objects and allowed to explore them freely for 3 min. The animals were then returned to their individual cages for a 15 min retention interval. After the elapse of this temporal interval, one of the objects was substituted with a new item. In the subsequent test phase (choice trial), every rat was reintroduced into the arena and allowed to explore both a familiar and an unfamiliar object. The rats were given 3 min to examine the objects, after which they were removed from the designated area and moved to their usual cages. The duration of each object investigation was noted when the rat’s head was pointed in the direction of the object at a distance of less than 2 cm [43].

Our results were analyzed in terms of the preference index (PI) and discrimination index (DI). The PI is the percentage of time spent exploring the novel object and was calculated as: PI = (*T*_N_/*T*_T_) × 100, where *T*_N_ represents the time spent with the novel object and *T*_T_ is the total exploration time for both objects. A PI greater than 50% indicates a greater preference for the novel object, whereas a PI less than 50% indicates a greater preference for the old object. The DI was calculated as: [DI = (*T*_N_− *T*_F_)/(*T*_N_+ *T*_F_)], where *T*_F_ represents the time spent exploring a familiar object. Inverted DI (negative DI) indicates a low exploration time for the novel object.

#### 2.4.2. MWM Test

The MWM is typically employed to evaluate spatial learning and memory through navigation‐based tasks that depend on hippocampal function. Training began 1 day after completion of the NOR test and consisted of 12 trials distributed over three consecutive days (four trials per day) [44]. During training, the hidden platform was positioned in the south‐east (SE) quadrant of the pool. As shown in Table 1, each rat started a 1 min trial from one of four different start points (north, west, south‐west, and north‐east), with a different order, every day. Rats were gently directed to the platform if they could not find it in the given time. In between trials, a 1‐h rest period was permitted. The platform was removed in order to perform a probe test on the fourth day. The rats started from the NW position for 1 min. The trials including the probe trial were recorded using ANY‐maze software. Performance was evaluated by measuring escape latency, quadrant time percentage, and path efficiency ratio during both training and probe trials. The escape latency is the time needed by the rats to reach the platform. The path efficiency is the ratio of a rat’s actual path length to the optimal path it could have taken to reach the target quadrant.

**Table 1:** Trial sequence and start positions for MWM.

Day	Trial sequence (starting positions)
1	N → W → SW→ NE
2	SW→ N → NE→ W
3	NE → SW → W → N
4	Probe trial (started at NW)

Abbreviations: N, north; NE, north‐east; NW, north‐west; SW, south‐west; W, west.

### 2.5. Biochemical Assays

#### 2.5.1. Colorimetric Assays

Colorimetric kits made by Biodignostics, Giza, Egypt, were used to quantify reduced glutathione (GSH) and malondialdehyde (MDA) and to measure the superoxide dismutase (SOD) activity. Every experiment was carried out with 10% hippocampal tissue homogenate in accordance with the manufacturer’s instructions. Results were calculated per milligram protein, after the total protein was quantified in each sample via a Bradford protein assay kit manufactured by BIO Basic, Inc., Canada [45].

#### 2.5.2. Enzyme‐Linked Immunosorbent Assay (ELISA)

The following markers were assayed using commercially available rat ELISAs: BDNF, IL‐1β (Elabscience, USA), p‐CREB (AFG Bioscience), total CREB (FineTest, China), doublecortin (DCX) (AFG Bioscience, USA), and caspase‐3 (Cusabio, USA). Following the manufacturer’s instructions, the sandwich ELISA technique was used for all experiments on a 10% hippocampus tissue homogenate. Like the colorimetric assays, the data are expressed per milligram of protein, quantified using the Bradford protein assay kit.

#### 2.5.3. Reverse Transcriptase‐Quantitative Polymerase Chain Reaction (RT‐qPCR)

Direct‐zol RNA Miniprep Plus (Zymo Research Co., USA) was used to deduce total RNA from the hippocampal tissue lysates. A Beckman dual spectrophotometer (USA) was then employed to evaluate the quantity and quality of RNA. Reverse transcription was performed via the Invitrogen SuperScript IV One‐Step RT‒PCR Kit (Thermo Fisher Scientific, Waltham, MA, USA). qPCR was performed in a single step via the prepared reaction mixture (Step One Applied Biosystem, Foster City, CA, USA). RNU6 and GAPDH were used as housekeeping genes for normalization of miR‐15a and ROCK‐1, respectively. The primers’ sequences are listed in Table 2.

**Table 2:** Sequences of primers used in the study.

Gene	Accession number	Sequence (5’‐3’)
RT‐primer	—	CTAGGCCTATTGATGGTGCCTACAG
*miR-15a*	XR_005648635.1	F: GCCGAGTAGCAGCACACATAA
		R: CAGTGCGTGTCGTGGAGT
*RNU6*	XR_006711274.1	F: GCTTCGGCAGCACATATACTAAA
		R: CGCTTCACGAATTTGCGTGTCAT
*ROCK-1*	NR_171200.1	F: AATCTTCCAGTTGGTTCTGCCT
		R: CTCTATTTGGTACAGAAAGCCAACC
*GAPDH*	NM_001394060.2	F: CCTTCTCCATGGTGGTGAAGA
		R: CACCATCTTCCAGGAGCGAG

The cycle threshold (Ct) was used to represent the data following the RT‒qPCR run. In each run, a control sample was used to gauge the expression of a particular gene. The Ct values of the evaluated genes were normalized to those of housekeeping genes, and relative quantification was performed using the delta‒delta Ct (ΔΔCt) computation via the 2^−∆∆Ct^ formula.

#### 2.5.4. Western Blotting

Western blotting was performed as previously described [12]. Briefly, proteins were extracted from hippocampal tissues, quantified using the Bradford assay, separated by SDS‐PAGE, and transferred onto membranes. After blocking, membranes were incubated with primary antibodies against p‐ERK1/2 (polyclonal; dilution 1/500, Abcam, ab214362) and ERK1/2 (polyclonal; dilution 1/500, Abcam, ab214362), followed by chemiluminescent detection (ChemiDoc MP imaging system (Bio‐Rad, 12003154)). Band intensities were normalized to β‐actin.

### 2.6. Histology and Immunohistochemistry

Rat brain tissue samples from various groups were promptly preserved in 10% formol saline for 72 h, after which they were processed to embedding in paraffin. Sections (4 μm) were stained with hematoxylin and eosin (H&E) to examine the overall morphology. Nissl (toluidine blue) staining was performed to identify neuronal integrity in the DG and CA3 regions. For Nissl staining, intact neurons have been identified as those showing a well‐defined cell body with a visible Nissl substance, a centrally located nucleus, and the absence of cytoplasmic vacuolization or pyknosis.

Immunohistochemical staining for Ki‐67 and ionized calcium‐binding adapter molecule‐1 (Iba‐1) was carried out using standard protocols, as previously described [12]. After 20 min of treatment with 0.3% H_2_O_2_, deparaffinized tissue slices were incubated with the primary antibody (anti‐Iba‐1 antibody, catalog # ab108539, dilution 1/100, Abcam, UK, or anti‐Ki‐67 monoclonal antibody, catalog # GTX16667, dilution 1/100, GeneTex Co., North America) incubated overnight at 4°C. Following 15 min of diaminobenzidine (DAB) treatment and rinsing, the samples were blocked with phosphate‐buffered saline, dehydrated, cleared in xylene, and then cover‐slipped for microscopic inspection. Sections were then incubated with appropriate secondary antibody (anti‐rabbit/mouse IgG, dilution 1:500; Vector Laboratories) and DAB for visualization.

To calculate the area‐based percentage of Ki‐67 and Iba‐1 immunostaining, six nonoverlapping high‐power fields (400x) were randomly chosen and scanned from the DG of each sample. Images were acquired using a Leica DM2500 light microscope (Leica Microsystems GmbH, Wetzlar, Germany) equipped with a Leica DFC450 digital camera, with identical exposure settings across all groups. Quantitative analysis was conducted employing the ImageJ software version 1.53K (National Institute of Health, USA). Positive staining was identified by cytoplasmic localization for (Iba‐1) and nuclear localization for (Ki‐67) with distinct chromogenic signals.

### 2.7. Statistical Analysis

GraphPad Prism 9.0.0 was used for all the statistical analyses. The mean ± standard deviation (SD) is used to express the data. Shapiro‒Wilk and Kolmogorov‒Smirnov tests were used to assess the normal distribution of variables. For training and probe escape latency, two‐way ANOVA test was employed. For the remaining variables, one‐way ANOVA followed by Tukey’s post‐hoc test was used to compare the study groups. Mead’s “resource equation” was utilized to ascertain whether the sample size was statistically sufficient, and Dixon’s Q test was employed to detect outliers. Adjusted *p*‐values were generated from Tukey’s HSD post‐hoc analysis. For every test, a *p*‐value < 0.05 was deemed statistically significant. Using 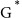
Power software version 3.1.9.7, a power analysis was used to calculate the group size (power = 0.9, *α* = 0.05).

## 3. Results

### 3.1. Combined Dex and Api Cotreatment Ameliorates the Behavioral and Cognitive Deficits Caused by MTX in Rats

**Figure 2:**
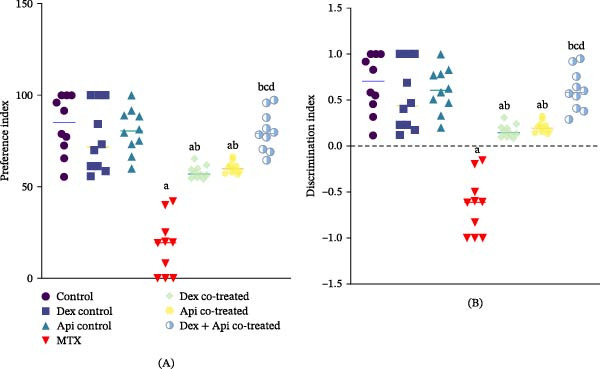
Effects of MTX, Dex, Api, and their combination on performance in the NOR test. (A) Preference Index (F (6,63) = 63.42, *p*  < 0.0001), (B) Discrimination Index (F (6,63) = 93.21, *p*  < 0.0001). The data are presented as means ± SD, *n* = 10. a Significant difference from the control; b, significant difference from MTX; c, significant difference from Dex cotreated group; and d, significant difference from Api cotreated group. *p*  < 0.05 indicates statistical significance. The data were analyzed via one‐way ANOVA followed by Tukey’s post‐hoc test.

The PI results indicated that MTX administration decreased rats’ preference for the novel object by 79.15% compared with the normal control group. Cotreatment with Dex or Api significantly increased the PI by 3.33‐ and 3.46‐fold, respectively, relative to the MTX group (*p*  < 0.05). Despite this improvement, PI values in both groups remained significantly lower than those in controls, with no significant difference observed between Dex and Api monotherapies. Notably, combined treatment with Dex and Api produced a significantly greater increase in PI compared with either monotherapy (*p*  < 0.05), suggesting a potential additive or synergistic effect in restoring recognition memory (Figure 2A).

Similarly, MTX administration caused a significant reduction in DI, decreasing it by 196.59%, and resulting in a negative DI ratio compared with the normal control. Cotreatment with Dex or Api significantly increased the DI compared with the MTX group, by 1.25‐ and 1.32‐fold, respectively. The combination of both cotreatments produced a significantly greater improvement in DI than either monotherapy (*p*  < 0.05), increasing DI by 1.93‐fold relative to the MTX group, which returned the values close to those of the control group (Figure 2B).

**Figure 3:**
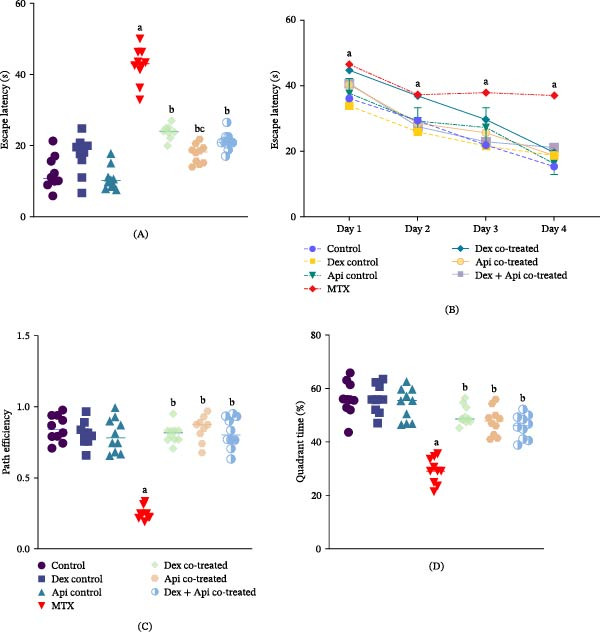
Effects of MTX, Dex, Api, and their combination on performance in the MWM test. (A) Escape latency (F (6,63) = 66.75, *p*  < 0.0001), (B) Escape latency during training trials and the probe test to evaluate the learning patterns of the rats over time, (C) Path efficiency ratio (F (6,63) = 66.75, *p*  < 0.0001), and (D) Quadrant time (F (6,63) = 66.75, *p*  < 0.0001). The data are presented as the mean ± SD, *n* = 10. a, Significant difference from the control; b, significant difference from MTX; and c, significant difference from Dex cotreated group. *p*  < 0.05 indicates statistical significance. (A, C, D) Data were analyzed using one‐way ANOVA followed by Tukey’s post‐hoc test. (B) Data were analyzed using two‐way ANOVA.

Spatial learning and memory were evaluated in the MWM by measuring escape latency. MTX‐treated rats exhibited a significant memory deficit, as indicated by a 3.55‐fold increase in escape latency relative to the control group. Cotreatment with Dex, Api, or their combination significantly reduced escape latency relative to the MTX group (*p*  < 0.05). Interestingly, Api cotreatment produced the greatest reduction in escape latency, decreasing it by 59% compared with the MTX group, whereas Dex cotreatment or Dex and Api combination reduced escape latency by 49% or 52%, respectively (*p* < 0.05) (Figure 3A). Additionally, analysis of the training performance over consecutive days (training trials and probe test) showed that the MTX group demonstrated impaired learning ability compared with that in the control and cotreated groups (*p* < 0.05) (Figure 3B).

Path efficiency was significantly reduced in MTX‐treated rats (~70.7%) compared to the controls. However, compared with those in the MTX group, the path efficiencies of the rats that received Dex cotreatment, Api cotreatment, and Dex + Api cotreatment were 3.26–, 3.4‐, and 3.3‐fold greater, respectively. However, no significant differences were observed among the three cotreated groups (Figure 3C).

In line with these findings, the percentage of time the rats spent in the target quadrant in the probe test was 48.15% lower in the MTX group than in the control group. Similarly, cotreatment with Dex, Api, or their combination increased the quadrant time by 1.71–, 1.62‐, and 1.57‐fold, respectively, compared with the MTX group. However, no significant difference was detected among the three cotreated groups (Figure 3D).

### 3.2. Combined Dex and Api Cotreatment Restored Hippocampal miR‐15a and Downregulated ROCK‐1 in MTX‐Treated Rats

**Figure 4:**
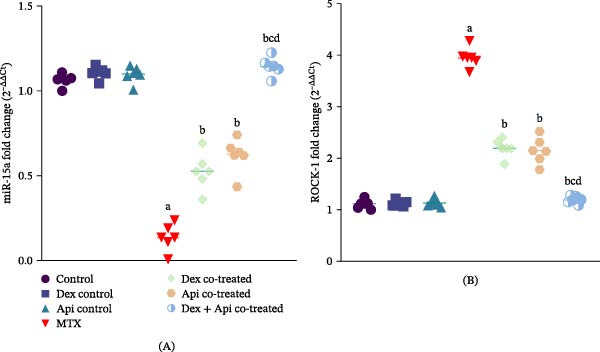
Effects of MTX, Dex, Api, and their combination on miR‐15a and ROCK‐1 expression. (A) miR‐15a (F (6,35) = 178.8, *p*  < 0.0001). (B) ROCK‐1, (F (6,35) = 296.1, *p*  < 0.0001). The data are presented as the mean ± SD, *n* = 6. a, Significant difference from the control; b, significant difference from MTX; c, significant difference from Dex cotreated; and d, significant difference from Api cotreated. *p*  < 0.05 indicates statistical significance. The data were analyzed using one‐way ANOVA followed by Tukey’s post‐hoc test.

As shown in Figure 4A, MTX administration significantly decreased the expression of miR‐15a in the hippocampus by 87.4% compared with that in the normal control group. In contrast, in the Dex‐ or Api cotreated groups, the expression of miR‐15a was 3.93‐ and 4.62‐fold higher than in the MTX group, respectively. Notably, the Dex + Api cotreated group showed a greater increase in miR‐15a expression than either drug alone, with an 8.5‐fold increase compared with the MTX group (*p* < 0.05).

In contrast, ROCK‐1 expression was 3.55‐fold greater in the hippocampi of MTX‐treated rats than in those of the control group (Figure 4B). The Dex‐ or Api cotreated groups showed significant reductions in ROCK‐1 expression of 44.26% and 45.58%, respectively, when compared with the MTX group. Combined Dex and Api administration produced a greater reduction in ROCK‐1 expression, decreasing it by 69.7% compared with the MTX group (*p*  < 0.05), indicating stronger modulation of the miR‐15a/ROCK‐1 axis than either monotherapy.

### 3.3. Combined Dex and Api Cotreatment Enhances Hippocampal ERK1/2 Activation

**Figure 5:**
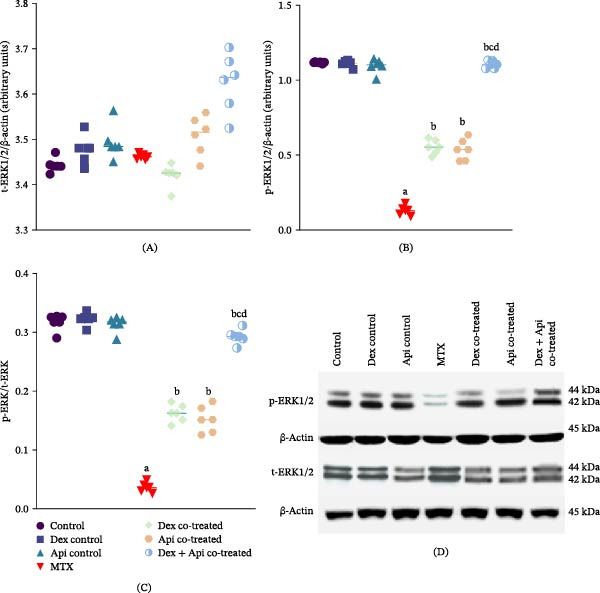
Effects of MTX, Dex, Api, and their combination on ERK1/2 activation. (A) t‐ERK1/2 (F (6,35) = 20.21, *p*  < 0.0001), (B) p‐ERK (F (6,35) = 595.2, *p*  < 0.0001), (C) p‐ERK/t‐ERK (F (6,35) = 374.8, *p*  < 0.0001), (D) Western blots for p‐ERK1/2, t‐ERK1/2, and β‐actin. The data are presented as the mean ± SD, *n* = 6. a, Significant difference from the control, b, significant difference from MTX, c, significant difference from Dex cotreated, and d, significant difference from Api cotreated. *p*  < 0.05 indicates statistical significance. The data were analyzed using one‐way ANOVA followed by Tukey’s post‐hoc test.

As shown in Figure 5, MTX administration markedly suppressed ERK1/2 activation in the hippocampus. Although MTX did not significantly affect total ERK1/2 (t‐ERK1/2) expression, it significantly reduced phosphorylated ERK1/2 (p‐ERK1/2) levels and the p‐ERK/t‐ERK ratio by 88.45% and 88.53%, respectively, compared with the control group (Figure 5A–D).

Compared with the MTX group, the Dex cotreatment significantly increased p‐ERK1/2 expression and the p‐ERK/t‐ERK ratio by 4‐ and 4.46‐fold, respectively. Similarly, the Api cotreatment significantly increased p‐ERK1/2 expression and the p‐ERK/t‐ERK ratio by 4.15‐ and 4.18‐fold, respectively, compared to the MTX group. Notably, Dex + Api cotreated group demonstrated a greater increase in ERK1/2 activation, resulting in 8.49‐fold and 8‐fold increases in p‐ERK1/2 expression and the p‐ERK/t‐ERK ratio, respectively, compared with the MTX group. This combined treatment produced a significantly greater ERK1/2 activation than either monotherapy (*p* < 0.05).

### 3.4. Combined Dex and Api Cotreatment Enhances Hippocampal CREB Activation and BDNF Expression

**Figure 6:**
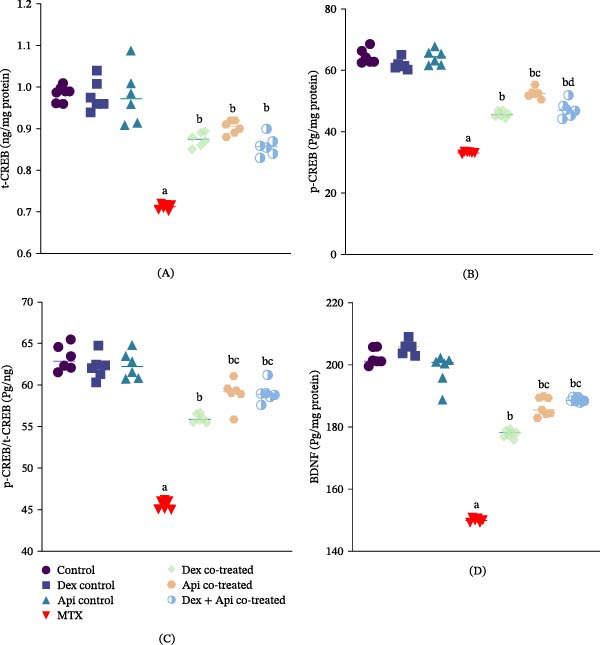
Effects of MTX, Dex, Api, and their combination on CREB activation and BDNF expression. (A) t‐CREB (F (6,35) = 55.95, *p*  < 0.0001). (B) p‐CREB, (F (6,35) = 219.2, *p*  < 0.0001). (C) p‐CREB/t‐CREB (F (6,35) = 137.0, *p*  < 0.0001). (D) BDNF (F (6,35) = 323.2, *p*  < 0.0001). The data are presented as the mean ± SD, *n* = 6. a, Significant difference from the control; b, significant difference from MTX; c, significant difference from Dex cotreated; and d, significant difference from Api cotreated. *p*  < 0.05 indicates statistical significance. The data were analyzed using one‐way ANOVA followed by Tukey’s post hoc test.

As shown in Figure 6A–C, MTX administration markedly suppressed CREB signaling in the hippocampus. Compared with the control group, MTX significantly reduced total CREB (t‐CREB), p‐CREB levels, and the p‐CREB/t‐CREB ratio by 28.1%, 48.66%, and 28.6%, respectively (Figure 6A‒C). Dex cotreatment partially restored CREB signaling, increasing t‐CREB and p‐CREB levels and the p‐CREB/t‐CREB ratio by 1.37‐, 1.22‐, and 1.23‐fold, respectively, compared with the MTX group. Similarly, Api cotreatment significantly enhanced CREB expression and activation, as indicated by increases in t‐CREB and p‐CREB levels and the p‐CREB/t‐CREB ratio of 1.26–, 1.58‐, and 1.30‐fold, respectively, compared to the MTX group. Of note, Api cotreatment resulted in significantly greater CREB phosphorylation relative to the Dex cotreated group (*p*  < 0.05). Although the Dex + Api combination significantly increased p‐ERK, it did not produce a synergistic enhancement of CREB activation. However, this combination increased t‐CREB and p‐CREB levels as well as the p‐CREB/t‐CREB ratio compared with the MTX group.

Consistent with the observed changes in CREB activation, BDNF expression was markedly reduced in the MTX group by 25.9% compared with the control group. Cotreatment with Dex, Api, or their combination significantly enhanced BDNF expression by 53.68%, 69.7%, and 73.9%, respectively, relative to the MTX group (*p*  < 0.05). Notably, Api and combined cotreatment produced comparable increases in BDNF expression, both of which were greater than that observed in the Dex cotreated group.

### 3.5. Combined Dex and Api Cotreatment Increased Hippocampal Cell Proliferation and Decreased Apoptosis in MTX‐Injected Rats

**Figure 7:**
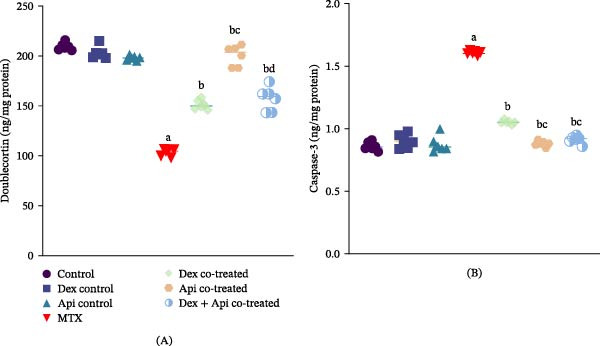
Effects of MTX, Dex, Api, and their combination on DCX and caspase‐3 levels. (A) Doublecortin (F (6,35) = 188,2, *p*  < 0.0001), (B) Caspase‐3 (F (6,35) = 299.1, *p*  < 0.0001). The data are presented as the mean ± SD, *n* = 6. a, Significant difference from the control; b, significant difference from MTX; c significant difference from Dex co‐treated, and d, significant difference from Api cotreated. *p*  < 0.05 indicates statistical significance. The data were analyzed using one‐way ANOVA followed by Tukey’s post‐hoc test.

Hippocampal neurogenesis was assessed by analyzing DCX expression, a marker of immature neurons and neuronal proliferation. As depicted in Figure 7A, DCX expression was 50.5% lower in the MTX group than in the control group, indicating impaired hippocampal neurogenesis. In contrast, in the Dex‐ and Api‐cotreated groups, the expression level of DCX was significantly increased by 1.45‐ and 1.93‐fold, respectively, compared with the MTX group (*p* < 0.05), with Api producing a significantly greater increase than Dex. However, the Dex + Api combination did not produce a further increase in DCX expression compared to Api cotreatment alone. This indicates no additional or synergistic enhancement of hippocampal neurogenesis in the Dex + Api combination compared with Api cotreatment alone.

Caspase‐3 was assayed to assess the apoptotic consequences of MTX treatment and the effects of Dex and Api cotreatment. MTX administration significantly increased caspase‐3 expression by 1.86‐fold compared with the control group (Figure 7B). Dex or Api cotreatment significantly decreased the caspase‐3 levels by 34.4% and 44.2%, respectively, compared with the MTX group (*p* < 0.05). The Dex + Api combination produced a comparable reduction in caspase‐3 expression (43.13% compared to the MTX group) to that observed with Api cotreatment, with both treatments showing more prominent effects than the Dex cotreatment group (*p* < 0.05).

### 3.6. Combined Dex and Api Cotreatment Mitigates Hippocampal Oxidative Stress and Neuroinflammation Caused by MTX in Rats

**Figure 8:**
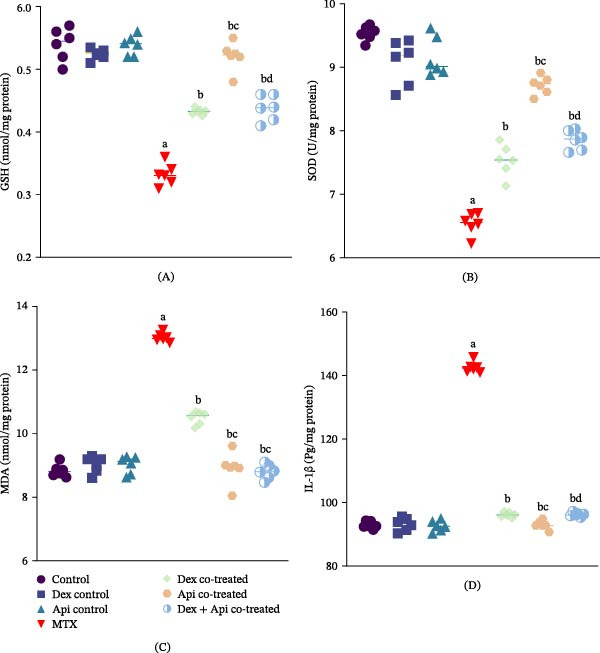
Effects of MTX, Dex, Api, and their combination on GSH, SOD, MDA, and IL‐1β. (A) GSH (F (6,35) = 112.2, *p*  < 0.0001), (B) SOD (F (6,35) = 78.84, *p*  < 0.0001), (C) MDA (F (6,35) = 186.0, *p*  < 0.0001), (D) IL‐1β (F (6,35) = 994.2, *p*  < 0.0001). The data are presented as the mean ± SD, *n* = 6. a, Significant difference from the control; b, significant difference from MTX; c, significant difference from Dex cotreated; and d, significant difference from Api cotreated. *p*  < 0.05 indicates statistical significance. The data were analyzed using one‐way ANOVA followed by Tukey’s post‐hoc test.

To assess oxidative stress in the hippocampus, GSH, and MDA levels and SOD activity were measured by spectrophotometry. MTX administration markedly increased oxidative stress in the hippocampus. MTX treatment significantly impaired oxidative status, as evidenced by significant reductions in GSH levels and SOD activity by 38.5% and 31.48%, respectively, along with a 1.47‐fold increase in MDA levels compared with controls (Figure 8A–C). Dex cotreatment attenuated the MTX‐induced oxidative stress by increasing GSH levels and SOD activity by 1.3‐ and 1.15‐fold, respectively, and reducing MDA level by 19.4% compared with the MTX group. Api cotreatment produced a significantly greater antioxidant effect than Dex, as evidenced by an increase in the GSH level and SOD activity of 1.56‐ and 1.33‐fold, respectively, as well as a decrease in the MDA level by 31.5% (*p*  < 0.05). However, the Dex + Api combination did not induce a further significant improvement in oxidative stress markers compared with Api cotreatment alone, indicating no additional or synergistic antioxidant effect.

Neuroinflammation was assessed by measuring IL‐1β levels in the hippocampus. MTX administration significantly increased IL‐1β levels by 1.53‐fold compared with the control group, indicating the neuroinflammatory consequences of MTX alone (Figure 8D). Dex and Api cotreatment decreased IL‐1β levels by 32.49% and 34.76%, respectively, compared with those in the MTX group. The Dex + Api combination produced a similar reduction in IL‐1β levels (32.48%) to that observed with Dex cotreatment, indicating no additional anti‐inflammatory effect of the combined treatment.

### 3.7. Combined Dex and Api Cotreatment Markedly Improved Hippocampal Neuronal Morphology in MTX‐Injected Rats

**Figure 9:**
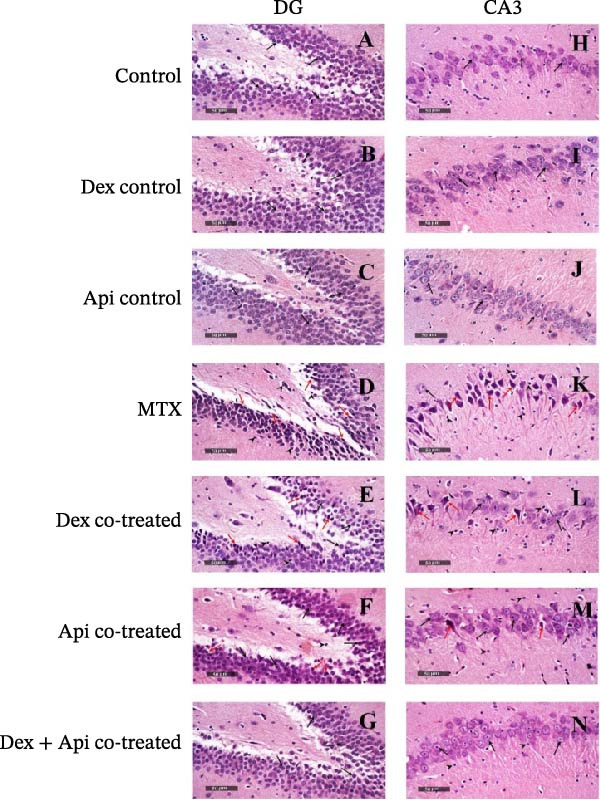
Hematoxylin and eosin (H&E) staining of the hippocampus of rats in different groups. (A–G) H&E staining of the DG. (H–N) H&E staining of CA3. (400× magnification).

Histological examination of the DG from the normal, Dex, and Api control groups revealed normal hippocampal architecture, including well‐organized granule cell layers with intact nuclear and subcellular details (black arrow), along with a normal hilar region without detectable abnormalities (Figure 9A–C). Sections from the DG of rats treated with MTX (Figure 9D) exhibited pronounced neuronal damage, characterized by numerous degenerated granule cells with pyknotic nuclei (red arrow), moderate edema, and increased glial cell infiltration (arrowheads). Dex cotreatment (Figure 9E) provided limited protection, as the histological features were comparable to those of the MTX group, with only a slight increase in the number of preserved granule neurons. The Api‐cotreated group (Figure 9F) demonstrated markedly greater neuroprotective efficacy, characterized by many apparently intact, well‐organized granule neurons with intact nuclear and subcellular details (black arrow); with occasional degenerated neurons (red arrow), and minimal reactive glial cell infiltrates. Interestingly, the Dex + Api combination (Figure 9G) resulted in almost intact morphological features of the DG, closely resembling those observed in the control groups.

Similarly, examination of the CA3 region revealed normal hippocampal morphology in the control groups (normal, Dex, and Api), with well‐organized pyramidal neurons and intact nuclear and cellular architecture (black arrow) (Figure 9H–J). In contrast, the MTX group (Figure 9K) showed severe neuronal degeneration characterized by an increased number of hypereosinophilic necrotic pyramidal neurons (red arrow), alternated with a few intact cells (black arrow), loss of distinct cellular details, mild perineuronal edema in the brain matrix, and markedly increased reactive microglial cell infiltration.

Dex cotreatment (Figure 9L) provided moderate protection, with a mixture of damaged neurons (red arrow) alternating with higher numbers of preserved neurons (black arrow) and milder glial cell infiltrates (arrowhead) compared with the MTX group. Api cotreatment (Figure 9M) showed significantly greater neuroprotective efficacy, with predominantly intact pyramidal neurons (black arrow), few sporadic degenerated or necrotic neurons (red arrow), and mild reactive glial cell infiltrates (arrowhead). Interestingly, the Dex + Api cotreatment group (Figure 9N) exhibited nearly normal CA3 architecture, with well‐preserved pyramidal neurons and minimal glial infiltration, closely resembling the histological appearance of the control groups.

**Figure 10:**
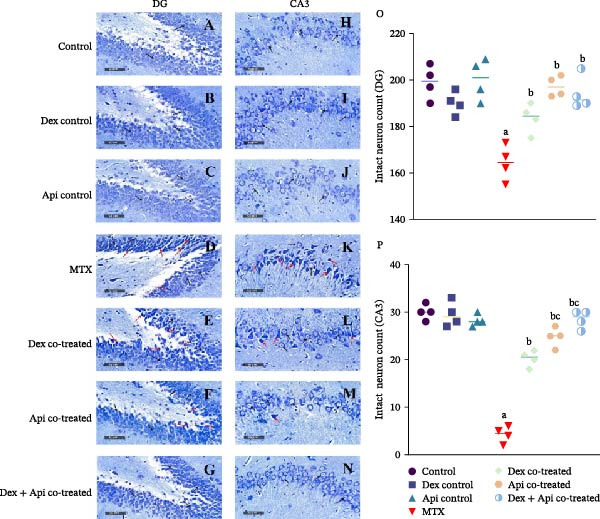
Nissl staining and quantitative analysis of intact neuron counts in the hippocampus of different experimental groups. (A–G) Nissl staining of the DG. (H–N) Nissl staining of CA3. (Red arrow) Damaged neurons (black arrow), intact neurons (400× magnification). (O) Intact neuron count (DG) (F (6,21) = 13.71, *p*  < 0.0001), (P) Intact pyramidal neuron count (CA3) (F (6,21) = 94.89, *p*  < 0.0001). The data are presented as the mean ± SD, *n* = 4. a, Significant difference from the control; b, significant difference from MTX; c, significant difference from Dex cotreated. *p*  < 0.05 indicates statistical significance. The data were analyzed using one‐way ANOVA followed by Tukey’s post‐hoc test.

Nissl staining was further performed in all the studied groups and examined by light microscopy at high magnification to determine the number of intact and degraded neurons in the DG and CA3 regions of the hippocampus (Figure 10A‒N). MTX administration significantly reduced the number of intact granule neurons in the DG region by nearly 17.46% (Figure 10O) and markedly decreased the density of large pyramidal cells in the CA3 region by 85.83% compared with those in the control group (Figure 10P). Conversely, Dex, Api, and Dex + Api cotreatment significantly increased the number of intact neurons in the DG region by 1.16–, 1.20‐, and 1.18‐fold, respectively, compared with the MTX group, which was comparable to that in the normal control group. No significant differences were observed among the three cotreated groups in the DG region.

Similarly, substantial increases in the number of intact large pyramidal neurons in the CA3 region were detected after treatment with Dex, Api, or their combination, with increases of 4.76–, 5.82‐, and 6.7‐fold, respectively, compared with the MTX group. Although the Dex + Api combination showed the greatest improvement in CA3 neuronal counts, the difference was not statistically significant compared with that of Api cotreatment alone (Figure 10O, P). These findings indicate that Dex and Api treatments effectively attenuated MTX‐induced neuronal injury, while the combination treatment did not produce a significantly additional benefit over Api alone.

### 3.8. Combined Dex and Api Cotreatment Enhances Hippocampal Neurogenesis and Attenuates Microglial Activation in MTX‐Injected Rats

**Figure 11:**
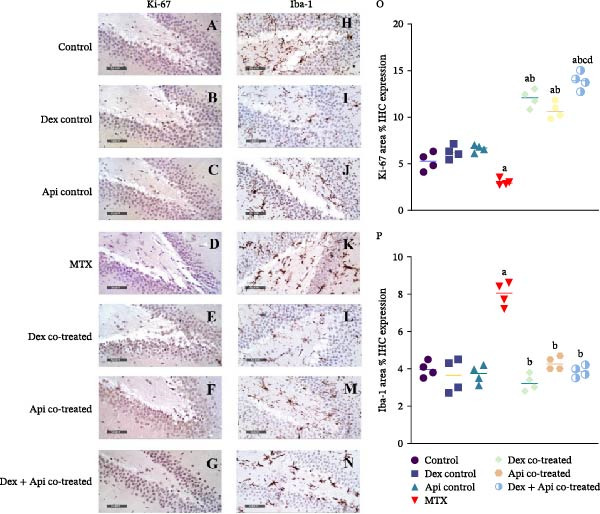
Effects of MTX, Dex, Api, and their combination on Ki‐67, a marker of cellular proliferation, and Iba‐1, a marker of microglial activation. (A–G) Representative immunohistochemical staining of Ki‐67 in the DG showing decreased Ki‐67 expression in the MTX group, whereas Dex‐, Api‐, and Dex+Api cotreated groups exhibited increased Ki‐67‐positive cells compared with the MTX group. (H–N) Representative immunohistochemical staining of Iba‐1 showing increased Iba‐1‐positive cells, indicating increased microglial activation in the MTX group compared with the normal group and reduced Iba‐1 expression in all the cotreated groups (400× magnification). (O) Ki‐67 area % IHC expression (F (6,21) = 102.9, *p*  < 0.0001), (P) Iba‐1 area % IHC expression (F (6,21) = 35.06, *p*  < 0.0001). The data are expressed as the mean ± SD, *n* = 4. a Significant difference from the control, b significant difference from MTX, c significant difference from Dex cotreated, and d significant difference from Api cotreated. *p*  < 0.05 indicates statistical significance. The data were analyzed using one‐way ANOVA followed by Tukey’s posthoc test.

Proliferative activity in the SGZ of the DG region was assessed by measuring the area percentage of Ki‐67‐positive cells (Figure 11A–G). MTX administration significantly decreased Ki‐67 expression by 44.58% compared with the control group, indicating impaired hippocampal proliferation. Conversely, Dex cotreatment significantly increased Ki‐67 expression, resulting in a 2.3‐fold increase compared with controls and a 4‐fold increase relative to the MTX group. Similarly, Api cotreatment significantly increased the percentage of Ki‐67‐positive cells by 3.56‐fold compared with the MTX group and 2.04‐fold compared with the control group. Notably, Dex + Api combination significantly increased the number of Ki‐67‐positive cells by nearly 2.65‐fold greater than that in the control group and 4.62‐fold higher than those of the MTX group (*p* < 0.05), indicating an additive or synergistic enhancement of hippocampal cell proliferation (Figure 11O).

Microglial activation was assessed by immunohistochemical staining of Iba‐1, as demonstrated in Figure 11H–N. MTX administration markedly increased the percentage of Iba‐1‐positive areas by 2.05‐fold compared with that in the control group, indicating increased microglial activation. On the other hand, Dex, Api, or their combination significantly decreased Iba‐1 expression by 59.24%, 51.72%, and 41.08%, respectively, compared with the MTX group (*p*  < 0.05). However, no significant differences were observed among the three cotreated groups, indicating comparable effects of Dex, Api, and their combination in attenuating microglial activation (Figure 11P).

## 4. Discussion

MTX is one of the most commonly used chemotherapeutic agents in the treatment of ALL, one of the most prevalent cancers among children. Although overall survival increases with time, children treated with MTX are reported to have neurotoxic side effects even after completion of chemotherapy. Increasing evidence suggests that MTX‐induced neurotoxicity is multifactorial, with impaired hippocampal neurogenesis and microglial activation emerging as important contributors. To the best of our knowledge, our previously published studies were the pioneer in analyzing the entire molecular mechanism underlying the MTX’s neurotoxic effect, demonstrating its effects on hippocampal neurogenesis and microglial activation. In addition, these studies are the first to show that Dex and Api can effectively mitigate MTX‐induced neurotoxicity in rats [12, 13]. However, the effect of Dex on microglial activation, the effect of Api on hippocampal neurogenesis, and the impact of their combination has not been fully studied. Hence, this study was performed to evaluate these effects and fill this research gap. This was accomplished by studying the effects of MTX alone, as well as of cotreatment with Dex and Api, and their combination, on the miR‐15a/ROCK‐1/ERK1/2/CREB/BDNF trajectory.

An evaluation of the cognitive function in the MTX group revealed that MTX substantially affected the spatial working memory and learning abilities in rats. In the NOR test, MTX‐treated rats had lower PIs and DIs than control rats, indicating recognition memory deficits. The results of the MWM test revealed that the MTX group failed to learn effectively to escape the maze, leading to increased escape latency, decreased quadrant time, and reduced path efficiency ratio in the probe test. These findings are consistent with previous studies reporting a decrease in hippocampal neurogenesis, with consequent cognitive impairment and memory deficits following MTX administration [10, 11, 43]. Interestingly, our findings showed that the combined Dex + Api treatment produced greater improvement on the NOR test but not on the MWM. This difference may be explained by the different cognitive domains evaluated by these two behavioral tests. NOR primarily assesses recognition memory that depends on the interactions between the hippocampus and the perirhinal cortex, making it highly sensitive to subtle changes in synaptic plasticity and neurogenesis [46, 47]. Therefore, the combined antioxidant, anti‐inflammatory, and neurogenic effects of Dex and Api may have produced a more pronounced improvement in recognition memory. In contrast, the MWM mainly assesses spatial learning and navigation, which rely on hippocampal spatial mapping and N‐methyl‐D‐aspartate (NMDA) receptor‐mediated synaptic plasticity [48]. Both Dex and Api are known to modulate NMDA receptor signaling. Dex has been reported to modulate NMDA receptor activity by inhibiting NR2B subunits and disrupting PSD95‐NMDA receptor interactions [49, 50], whereas Api exerts neuroprotective effects partly through antagonizing NMDA receptors and reducing excitotoxicity [51]. Therefore, restoration of NMDA receptor‐dependent synaptic plasticity by either treatment alone may have been sufficient to improve spatial learning in the MWM, which could explain why the combined treatment did not produce additional improvement compared with monotherapy.

The present biochemical and histochemical results, along with the behavioral data from the NOR test and MWM, revealed marked MTX‐induced neurotoxicity via impairment of neurogenesis and activation of microglia in the hippocampus through alterations in the miR‐15a/ROCK‐1/ERK1/2/CREB/BDNF trajectory. All of these findings suggest that neuroprotective cotreatment is needed to attenuate the neurotoxic effects of MTX, especially since this effect can be aggravated by the downregulation of miR‐15a reported in different types of leukemia and lymphoma [52].

Dex and Api cotreatments were studied as possible attenuators of MTX‐induced neurotoxicity. To study cognitive function and memory, the NOR test and MWM test were performed. In the NOR test, both Dex and Api improved the PI and DI with similar magnitudes. However, their combination was more effective than either monotherapy alone in increasing the PI and DI. In the MWM test, all the cotreated groups presented a nearly linear decline in escape latency over the training days, indicating an improvement in the learning ability of the rats. However, in the probe test, the Api cotreatment markedly decreased the escape latency more prominently than the Dex or the combined treatment did. The path efficiency ratio and quadrant time increased in all the cotreated groups in a nearly equal manner. The Dex favorable effect on cognitive behavior may be explained in part by its ability to promote oligodendrocyte genesis and myelination following hypoxic‐ischemic brain injury in neonatal rats [39]. Api administration restored myelin basic protein levels in a rat model of methylmercury‐induced neurotoxicity, indicating the positive impact on oligodendrocyte function and myelin protection [53]. We also discussed that Dex and Api combined regimen has anti‐inflammatory and antioxidant effects, as indicated in our study, which could indirectly support oligodendrocyte survival and myelin maintenance. Altogether, Api could be a complementary agent in combination therapies targeting neurotoxicity.

In the present study, miR‐15a expression was reduced in the hippocampi of MTX‐treated rats. These results support previously published data that a decrease in miR‐15a is associated with AD [34]. Using the Pathway Studio online tool, a bioinformatics analysis revealed a complex interaction network between miR‐15a, ROCK‐1, ERK1/2, and CREB [13]. These molecules are known to play a role in regulating the neurogenesis process, and they are linked to BDNF. These results suggest that miR‐15a directly suppresses ROCK‐1 while simultaneously promoting ERK1/2 activity. Activated ERK1/2 has favorable regulatory effects on CREB and BDNF, leading to increases in neurogenesis, cell survival, neuroplasticity, and anti‐inflammatory effects. Our results revealed that changes in miR‐15a expression were associated with modulation of the ROCK‐1/ERK1/2/CREB/BDNF axis and may contribute to neuronal survival.

ROCK‐1 was increased downstream of miR‐15a in the MTX group. Prior research have connected ROCK‐1 upregulation to various neurodegenerative disorders, including AD [32]. ROCK‐1 upregulation leads to significant deactivation of the neuroprotective ERK1/2/CREB/BDNF pathway. These results confirm the findings of previously reported studies that illustrated the ROCK‐1/ERK1/2 inhibitory cross‐interaction. These studies reported increased ERK1/2 activity in ROCK inhibitor‐treated models [54, 55]. In addition to the previously published data, ROCK‐1 inhibition activates ERK1/2 and promotes adipogenesis [56]. In the present study, ERK1/2 activation, as measured by phosphorylation at Ser133, was decreased, leading to a significant decrease in CREB expression and its activation via phosphorylation at Ser133. The inactivation of CREB significantly decreased BDNF expression.

We observed that MTX administration induced substantial deterioration in the DG and CA3 regions of the hippocampus. These changes included severe loss of neuronal tissue as well as a marked reduction in the size of large pyramidal neurons. Immunohistochemical analysis revealed a considerable increase in Iba‐1 immunostaining, accompanied by reduced Ki‐67 expression in the hippocampus of MTX‐treated animals. The increased Iba‐1 staining, together with prominent glial cell infiltration in the DG and CA3 regions of the hippocampus, reflects increased microglial activation. This microglial activation can be attributed to the direct effect of ROCK‐1 overexpression. These observations are in consistent with the previous reports linking MTX exposure to microglial activation [17, 57, 58].

Immunohistochemical examination of Ki‐67 revealed that its expression in the DG and CA3 regions of the brains of the MTX group decreased considerably, indicating impaired hippocampal neurogenesis. In addition, DCX levels, a critical component of neurogenesis and a hallmark of immature neurons, were markedly reduced following MTX treatment compared to the control group. This reduction suggests that MTX‐induced neurotoxicity may result from compromised neurogenesis, reduced cell proliferation, and increased microglial activation.

ROCK‐1 activation has been associated with increased production of inflammatory cytokines in the hippocampus, which may contribute to neuronal damage and cognitive impairment [33]. A decrease in BDNF, along with microglial activation, is known to induce oxidative stress, neuroinflammation, and apoptosis, leading to various neurodegenerative diseases [26–29]. In the MTX group, ROCK‐1 was upregulated, BDNF was downregulated, and microglia were activated, indicating increased hippocampal oxidative stress. This can also be a result of the increase in the inflammatory mediator IL‐1β, leading to apoptotic activity, as evidenced by elevated caspase‐3 levels. These findings support earlier studies associating neuroinflammation and reduced BDNF levels with hippocampal cognitive impairment [59].

Dex has shown interesting cardioprotective, anti‐inflammatory, and neuroprotective effects as a highly selective α2‐adrenergic agonist [60]. In the present study, Dex attenuated MTX‐induced alterations and was associated with increased miR‐15a expression and subsequent downregulation of ROCK‐1. Increasing evidence suggests that ROCK‐1 downregulation may modulate microglial activation and activate the ERK1/2/CREB/BDNF neuroprotective pathway [61], consistent with earlier findings in cardiac muscle cells and NCM460 cells models [53, 62].

Iba‐1 immunostaining is sensitive, widely used marker to detect microglia and to estimate microglial activation based on morphological alterations and increased immunoreactivity [63]. In this study, we showed that Dex or the Api+Dex had a favorable impact on microglial activation using a well‐known marker such as Iba‐1. Mechanistically, ROCK‐1 downregulation in the Dex‐treated group was associated with decreased Iba‐1 immunostaining compared with the MTX group. This reduction of ROCK‐1 may also be linked to activation of ERK1/2/CREB/BDNF signaling pathway. The Dex‐cotreated group presented marked activation of ERK1/2, as shown by increased p‐ERK1/2 and p‐ERK/t‐ERK. Downstream of ERK1/2, CREB expression, as well as its phosphorylation, increased. When CREB is active, it activates the expression of BDNF. In the Dex cotreated group, Dex was able to retain the lost BDNF in the MTX group. According to Tu et al., Dex activates ERK1/2/CREB/BDNF, increasing p‐ERK1/2, p‐CREB, and BDNF protein expression [61]. Furthermore, inhibition of ERK1/2 and CREB signaling abolished the neuroprotective effect of Dex, supporting the involvement of this pathway [38].

BDNF is crucial for cell proliferation and viability. The present findings demonstrated that Dex significantly increased Ki‐67‐positive cells and elevated DCX expression in the hippocampus of rats treated with MTX compared with controls. The observed increase in cell proliferation can be explained by the findings of previous studies, which reported that Dex promotes cell proliferation through multiple molecular pathways. It has been shown that Dex increased phosphorylated phosphoinositide‐3‐kinase (p‐PI3K) and phosphorylated protein kinase B (p‐AKT) levels, key regulators of cell survival and proliferation. Additionally, Dex downregulates the long noncoding RNA LINC00982, which negatively regulates cell growth, thereby facilitating a pro‐proliferative environment [64]. Another study reported that Dex enhances cell proliferation and viability in LPS‐induced models, indicating its role in modulating inflammatory and neurogenic responses [65]. Additionally, the increase in BDNF and the decrease in microglial activation improved the inflammatory and redox status of the hippocampus. Compared with the MTX‐treated group, Dex treatment was associated with reduced IL‐1β and MDA levels and improved GSH levels and SOD activity. These findings are supported by published data concerning the protective effect of Dex injury in vascular smooth muscle and lungs through the amelioration of oxidative stress [66–68]. Collectively, these effects were associated with reduced apoptosis, as indicated by decreased caspase‐3, and increased numbers of intact neurons in the DG and CA3 regions.

Notably, Api monotherapy produced greater improvements in DCX and GSH levels and SOD activity compared to combined treatment. Similar to Dex, Api alleviated MTX‐induced neurotoxicity by modulating the miR‐15a/ROCK‐1/ERK1/2/CREB/BDNF pathway, thereby reducing neuroinflammation and apoptosis, restoring redox balance, improving hippocampal neurogenesis, and mitigating microglial activation in the rat hippocampus. These beneficial effects were accompanied by improvements in cognitive function, highlighting its neuroprotective effects. The stronger effect of Api on DCX, GSH, and SOD levels may reflect its potent antioxidant and neurogenic properties in the present model, particularly with respect to redox balance and expression of immature neuronal markers [69]. Because Dex and Api may converge on partially overlapping protective pathways, the addition of Dex may not necessarily amplify all downstream effects of Api [38, 70, 71]. Moreover, Api treatment alone may have already produced a near‐maximal response in these parameters, limiting the ability of cotreatment to produce further improvement. The difference between Ki‐67 and DCX responses may also be explained by the fact that these markers reflect distinct stages of neurogenesis, with Ki‐67 indicating proliferative activity and DCX reflecting immature neuronal differentiation [72–74].

Compared with MTX treatment, Api cotreatment augmented miR‐15a, while reduced ROCK‐1 expression. The neuroprotective effect of ROCK‐1 downregulation was evidenced by decreased microglial activation, thus decreasing Iba‐1 expression. These findings support previously published data highlighting the anti‐inflammatory effects of Api in cultured microglia and models of AD [75, 76]. In addition, ROCK‐1 downregulation may contribute to activation of the ERK1/2/CREB/BDNF signaling pathway. This was supported by increases in the p‐ERK/t‐ERK and p‐CREB/t‐CREB ratios, suggesting enhanced pathway activity. When CREB was more highly phosphorylated, BDNF expression was greater than that in the MTX group. These data are in line with previously mentioned findings that BDNF is promoted in Api‐treated mice in a scopolamine‐induced cognitive dysfunction experimental model [77].

Api is well known to have antioxidant and anti‐inflammatory properties [40, 71]. By inhibiting microglial‐induced inflammation and increasing BDNF expression, Api decreases IL‐1β, MDA, and caspase‐3 levels. Api also increased both GSH levels and SOD activity, thus maintaining a healthy redox balance. These results are in accordance with previous reports describing its antioxidant, anti‐apoptotic, and neurogenic effects [78–80]. Api not only improved microglial activity and inflammatory markers but also markedly improved the impaired neurogenesis caused by MTX. Both Ki‐67 and DCX significantly increased in the Api‐cotreated group when compared with the control group. Furthermore, it obviously increased the neuronal count in the DG and CA3 regions, suggesting its positive impact on hippocampal neurogenesis. This is consistent with a previous study, which reported that Api enhances neurogenesis by promoting cell proliferation and differentiation through the activation of the ERK/CREB/BDNF signaling pathway [12]. Additionally, Api binds directly to BDNF, stimulating its neurotrophic activities, which include increased neuronal survival [81]. Interestingly, the effects of Api on DCX expression, redox status, and apoptosis were more pronounced than those of Dex. This could explain the greater improvement in hippocampus histopathology in the Api‐cotreated rats than in the Dex‐cotreated rats.

Since both Dex and Api were individually effective, the current study investigated whether their combination would provide greater benefit than either monotherapy. The combined treatment produced more pronounced improvements in certain outcomes, including recognition memory in the NOR test, miR‐15a and ROCK‐1 expression, ERK1/2 activation, Ki‐67 expression, and qualitative histopathological appearance. However, this enhanced effect was not observed uniformly across all assessed parameters. The combination did not provide additional benefit over Api alone in the MWM, DCX expression, oxidative stress markers, apoptosis, or BDNF expression, and it had effects comparable to monotherapy on Iba‐1 immunostaining and IL‐1β levels. Notably, the enhanced effect of the combined treatment was primarily observed at the upstream levels of the miR‐15a/ROCK‐1/ERK1/2/CREB/BDNF pathway, including increased miR‐15a expression, ROCK‐1 inhibition, and ERK1/2 activation. In contrast, CREB phosphorylation and BDNF expression were not further increased compared with Api monotherapy. These findings suggest that this combination exerts greater effects in specific domains, particularly recognition memory, ERK‐related signaling, and proliferative activity, rather than across the entire signaling cascade.

Furthermore, histopathological analysis of the hippocampus revealed a remarkable improvement in the DG region and a marked increase in intact CA3 neurons in combination‐treated rats compared with the MTX group, with values comparable to those observed in the Dex cotreated and Api cotreated groups. The combination also produced a greater increase in Ki‐67 expression than monotherapy, indicating enhanced cell proliferation and neurogenesis. In contrast, the combination had no significant effect on Iba‐1 immunostaining compared with the other cotreated groups.

The current study has several limitations. First, as this was an animal experimental study, the dosing regimen and dose calculations may differ from those used in clinical settings, although the routes of drug administration used in this study mimic clinically relevant approaches. Second, the molecular findings are associative and do not establish direct causality within the miR‐15a/ROCK‐1/ERK1/2/CREB/BDNF trajectory, as functional manipulation experiments such as miR‐15a or ROCK‐1 knockdown or overexpression were not performed. The absence of locomotor control measurements for the Dex sedative effect is another limitation. However, the degree of sedation caused by Dex varies with dose, and the used dose in this study (10 µg/kg) is considered low with a short‐term (15–30 min) and reversible effect [82, 83]. In addition, behavioral assessments were conducted by the end of the study period, which likely minimized this potential confounding factor. Third, microglial activation was assessed using Iba‐1 immunostaining alone, which reflects overall microglial activation but does not distinguish between specific activation phenotypes. Fourth, hippocampal neurogenesis was evaluated using DCX expression and Ki‐67 immunostaining, but quantification of DCX‐positive cells by immunohistochemistry was not performed. Such analysis would provide a more precise spatial assessment of hippocampal neurogenesis and should be considered in future studies. Moreover, molecular analyses were performed on whole hippocampal homogenates, which limits cell‐specific interpretation of the observed pathway alterations. In addition, the effects of MTX, Dex, and Api on brain regions other than the hippocampus, as well as on other relevant neural cell populations, such as oligodendrocytes, were not examined. Future studies that address these limitations and evaluate the potential systemic toxicities of these treatments would further refine the mechanistic interpretation and translational relevance of the present findings. Further clinical studies are warranted to validate these findings.

## 5. Conclusion

In conclusion, the combination of Dex and Api as cotreatments with MTX improved selected behavioral, molecular, and histological parameters in MTX‐induced neurotoxicity. The Dex + Api combination produced greater or comparable effects than either monotherapy in recognition memory, modulation of the miR‐15a/ROCK‐1 axis, ERK1/2 activation, and Ki‐67 expression. The combination did not provide additional benefit over Api alone in the MWM, DCX expression, oxidative stress markers, apoptosis, or CREB/BDNF signaling. Furthermore, this combination has effects comparable to Api or Dex monotherapy on Iba‐1 immunostaining and IL‐1β levels. These findings suggest that the combined treatment may provide enhanced neuroprotection in specific domains, while its benefits are not uniform across all assessed parameters.

## Author Contributions

Conceptualization: Omar Mohsen Eldemerdash, Mahmoud A. Senousy, Mohamed Taha and Ismail Mohamed Elshaffei; Data curation, Einas M. Yousef, Omar Mohsen Eldemerdash, Mahmoud A. Senousy and Haidy Mohammed. Formal analysis: Einas M. Yousef, Omar Mohsen Eldemerdash, Mahmoud A. Senousy, Mohamed Taha, Haidy Mohammed and Ismail Mohamed Elshaffei. Funding acquisition: Einas M. Yousef, Omar Mohsen Eldemerdash and Mohamed Taha. Investigation: Einas M. Yousef, Omar Mohsen Eldemerdash, Mahmoud A. Senousy, Mohamed Taha, Haidy Mohammed and Ismail Mohamed Elshaffei. Methodology: Einas M. Yousef, Omar Mohsen Eldemerdash, Mahmoud A. Senousy and Haidy Mohammed. Resources: Omar Mohsen Eldemerdash and Mahmoud A. Senousy. Supervision: Mohamed Taha and Ismail Mohamed Elshaffei. Validation: Einas M. Yousef, Mahmoud A. Senousy, Mohamed Taha and Ismail Mohamed Elshaffei. Visualization: Einas M. Yousef, Omar Mohsen Eldemerdash, Mahmoud A. Senousy and Mohamed Taha. Writing – original draft: Einas M. Yousef, Omar Mohsen Eldemerdash, Mahmoud A. Senousy and Haidy Mohammed. Writing – review & editing: Einas M. Yousef, Omar Mohsen Eldemerdash, Mahmoud A. Senousy, Mohamed Taha, Haidy Mohammed and Ismail Mohamed Elshaffei. Writing – finalize: Einas M. Yousef, Omar Mohsen Eldemerdash, Mahmoud A. Senousy, Mohamed Taha, Haidy Mohammed and Ismail Mohamed Elshaffei.

## Funding

This research received no external funding, and the APC was funded by Alfaisal University. Riyadh, Saudi Arabia.

## Disclosure

All authors have read and agreed to the published version of the manuscript. All content was carefully reviewed and validated by the authors, who take responsibility for the final manuscript.

## Ethics Statement

The animal study protocol was approved by the Research Ethics Committee for Experimental and Clinical Studies, Faculty of Pharmacy, Cairo University, Cairo, Egypt (Permit Number BC2944).

## Consent

The authors have nothing to report.

## Conflicts of Interest

The authors declare no conflicts of interest.

## Data Availability

The data that support the findings of this study are available from the corresponding author upon reasonable request.
